# The optimal movement patterns for mating encounters with sexually asymmetric detection ranges

**DOI:** 10.1038/s41598-018-21437-3

**Published:** 2018-02-20

**Authors:** Nobuaki Mizumoto, Shigeto Dobata

**Affiliations:** Laboratory of Insect Ecology, Graduate School of Agriculture, Kyoto University, Kitashirakawa-oiwakecho, Sakyo-ku, Kyoto 606-8502 Japan

## Abstract

Animals have evolved various sex-specific characteristics to improve the efficiency of mating encounters. One is the sex-specific attracting signal. Signal receivers perform a combination of random search and navigation before and after signal detections. On the other hand, signal senders can also modify their movement patterns to optimize their encounter rates, which invokes a reverse side of random search problems that asks for the most efficient movement patterns of signal senders to be found by signal receivers. In this study, we focused on visual and auditory signals in particular, and quantified the efficiency of mating encounters of individual animals performing a Lévy walk, a special class of random walk, with a variety of speeds before signal detection. We found that signal senders should move more slowly and/or less diffusively than receivers to improve mating encounters. The optimal movement patterns of senders ranged from relatively slow to stationary ones depending on the density of individuals, the effective range of signals, and the ability of receivers to locate senders. By focusing on the optimal movement patterns of individuals that are often assumed to be given targets, the present study provides insights into strategies of effective attraction beyond the case of mate search.

## Introduction

Organisms exhibit movements to encounter other organisms that can be prey, hosts, or mating partners^1,2^. When searchers have no or little information about the locations of their targets, searchers will perform random search as a strategy to efficiently encounter targets^3^. Considering different random search strategies can result in different encounter rates, optimal foraging theory predicts that searchers show the movement patterns that maximize the encounter rate, and thus their fitness^3,4^.

The movement patterns of animals sometimes exhibit a scale-free characteristic, and are called Lévy walks^2,5–12^. Lévy walks have attracted a great deal of attention because of their advantage among random search strategies to encounter targets that are randomly and sparsely distributed. The Lévy walk is a special class of random walk models in which the probability density function of step length *l* has a power-law tail: $P(l) \sim {l}^{-{\rm{\mu }}}$ (1 < μ ≤ 3), where μ is a power-law exponent^13^. For µ ≥ 3, the movements correspond to a Brownian walk owing to the central limit theorem, whereas µ ≤ 1 is not a normalizable probability distribution^13^. The Lévy walk includes a variety of random walk processes from ballistic motion (Lévy exponent μ → 1) to Brownian random walk (Lévy exponent μ = 3). Previous studies have thoroughly analyzed the efficiency of a variety of Lévy walk patterns under various conditions, including the availability of targets^13–15^, their movement patterns^16–18^, the ecological interactions^19^, and searching environments^15,20^. However, since most of these studies only considered the optimal movement patterns for searchers to gain targets with given distributions or movements, there is still room to consider the viewpoint from targets which can also modify their movement patterns for their own benefit. In a mate search, for example, an encounter can be beneficial for both males and females. Thus, one should consider not only optimization by one sex to the given mating partners but also mutual optimization by males and females in the case of a random search problem for mating encounters^21^.

Moreover, in mate search, males and females have developed sex-specific characteristics to improve the efficiency of mating encounters. Mate search is often mediated by sex-specific attracting signals, by which individuals of one sex indicate their presence and location of themselves to the other sex^22^. Signals can be auditory (calling or vibration^23–25^), visual (coloration or flashing^18,26,27^) or chemical (pheromones^28,29^). The presence of these sex-specific attracting signals leads to sexual differences in perceptual distances, that is, signal receivers can detect the signal senders from a more distant position than signal senders can detect the receivers.

In mate search with attracting signals, the movements of individuals can be categorized by two phases according to their relation to signal detection. One is the “random walk” without signals, where individuals are assumed to look for mating partners without any knowledge of their possible locations^14,29^. The other is the “navigation behavior” after signal detection, where individuals that have received signals change their behaviors to be guided directly or indirectly by the source of the signal^29–31^. Signal receivers would perform combinations of random searches and navigation behaviors before and after signal detection, respectively, whereas signal senders would perform only random searches while emitting signals, reflecting their much smaller perception range than receivers. It has been studied how signal receivers optimize their random walk strategies to find signals^18,32^ and how they orient and locate the source of signals by their navigation mechanisms^30^. In contrast, although signal senders can also modify their movement patterns to optimize their encounter rates, how they achieve this has rarely been considered. This invokes a reverse side of random search problems that asks for the most efficient movement patterns of signal senders to be found by signal receivers.

The availability of signals can vary depending on the types of signals^33,34^. Visual or acoustic signals are available instantaneously around signal senders, while chemical signals are often subject to a prevailing wind (flow) direction or turbulence. In this study, we focused on mate search with attracting signals, where senders are assumed to emit signals continuously and receivers can perceive them instantaneously. Such a characteristic would be fulfilled by a specific coloration in visually cued mate search or by flashing, calling and vibration that are emitted periodically. By developing a simulation model, we quantified the efficiency of mating encounters of monogamous individuals performing a Lévy walk with attracting signals. The efficiency was measured as the number of pairs formed within a given time in a population. This measurement is proportional to the efficiency of both receivers and senders, and is convenient to show the efficiency for mutual search. Note that maximizing the number of pairs formed within a given time is equivalent to minimizing the time required to encounter a mating partner (mean first passage time: MFPT). MFPTs are expected to be the same between receivers and senders with a sufficient long time. We modeled movements of receivers and senders by the Lévy walks with 1.1 ≤ µ ≤ 3.0 because it can easily represent a rich variety of movements with different diffusivity.

We first explored the optimal combinations of movement patterns of receivers and senders when their moving speeds were equal. Both the results of the current study and the theory of random search strategies predict that receivers always favor to perform ballistic motion at as fast a speed as possible when targets are destructive (i.e., disappear after mating) and are available all over the searching space (e.g., periodic boundary conditions)^17^. This is because the primary goal for receivers is to detect the signals, and the most efficient course of action is then to cover as much ground as possible by avoiding already-visited places^17^. On the other hand, for the movement patterns of senders, it can be expected that (1) senders may need to avoid already-visited places so as to be statistically likely to come near a receiver before detecting the signal, and (2) senders may need to stay in the same place so that receivers can easily locate them after detection. The former favors the ballistic motion with small µ value and the latter favors the movements with large µ or even no movements. Thus, we then examined the most efficient movement patterns for senders to encounter the ballistic receivers with the presence of attracting signals.

## Results

**Figure 1:**
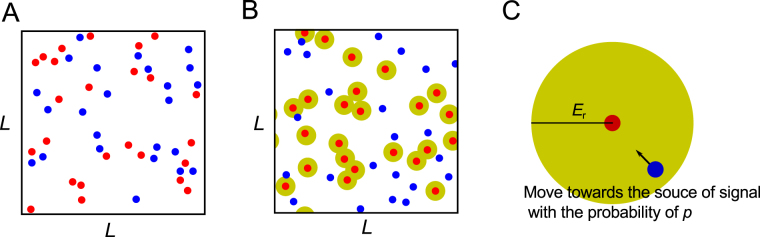
A depiction of the simulations. Signal receivers (blue circles) and senders (red circles) search for each other in a two-dimensional space with periodic boundary conditions (size: *L*^2^) without (**A**) or with attracting signals (**B**). Senders have an effective attraction radius (*E*_r_) of attracting signals, within which receivers are oriented towards the senders with a probability of *p* at each time step (**C**).

We developed a simulation model to examine the efficiency of mating encounters of individuals performing a Lévy walk with attracting signals. In the simulations, *n* males and *n* females were assumed to be searching for the opposite sex in a two-dimensional space with periodic boundary conditions (size: *L*^2^; Fig. 1A,B). In this study, we denote two sexes by “receivers” and “senders,” where senders have sex-specific signals for attracting receivers. We only assumed that senders and receivers belong to different sexes. The attracting signal was assumed to be effective around signal senders (called signal effective space), with receivers only responding to the signals after entering the effective space. For simplicity, we described this signal effective space as the effective attraction radius (EAR) to model the attraction of visual or acoustic signals. We assumed that senders have EARs denoted by *E*_r_ from the center of their body, within which receivers move towards the senders with a probability of *p* (i.e., they are attracted) and random direction with (1 − *p*) (i.e., they lose the signal) in each time step (Fig. 1C). Thus the *p* was considered as the ability of receivers to locate senders (locatability).

**Figure 2:**
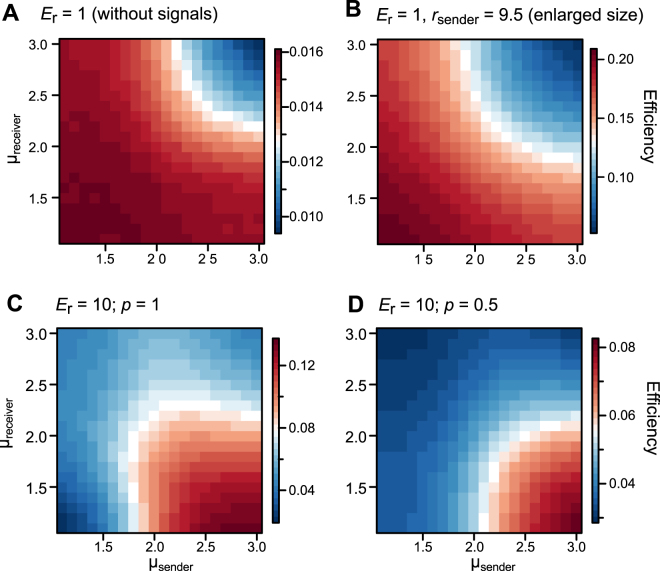
The simulation results performed with receivers and senders with the same moving speed (*v*_receiver_ = *v*_sender_ = 1; *L*^2^ = 100,000) across various conditions. (**A**) The absence of attracting signals, where the encountering distance is the same as the size of effective attraction radius (*E*_r_ = 1). (**B**) The absence of attracting signals with the size of senders (*r*_sender_) enlarged (*r*_sender_ = 9.5). (**C**) The presence of attracting signals with a high locatability of receivers (*E*_r_ = 10; *p* = 1). (**D**) The presence of attracting signals with a low locatability of receivers (*E*_r_ = 10; *p* = 0.5). The efficiency is computed as the average number of pairs encountered at a step.

In the absence of attracting signals, a sexually monomorphic population with ballistic motion (μ = 1.1) achieved the highest encounter rates (Fig. 2A). On the other hand, in the presence of attracting signals, a sexually dimorphic population with receivers with ballistic motion (μ = 1.1) and senders with Brownian walk (e.g., μ = 3.0) achieved the highest encounter rates when the speeds of movement were the same between sexes (Fig. 2C,D). The optimal combinations of receivers’ and senders’ movement patterns were qualitatively similar irrespective of the locatability of receivers, the density of individuals, and the size of effective attraction radius (Fig. 2C,D; Supplementary Figures S1, S2). The optimal movement patterns of receivers were always ballistic (μ = 1.1) across parameters (Fig. 2; Supplementary Figures S1, S2). Enlarging senders’ size did not qualitatively affect the results without attracting signals (Fig. 2B).

**Figure 3:**
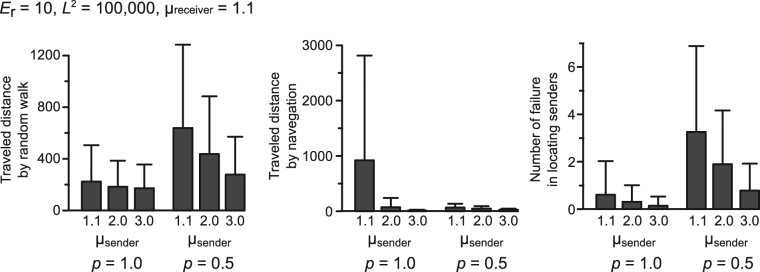
The search process by receivers (μ_receiver_ = 1.1) to find senders in mate search with attracting signals (*v*_receiver_ = *v*_sender_ = 1; *L*^2^ = 100,000). The search process was divided into random walk before detecting signals and navigation behavior in EAR of signal senders. If receivers failed in locating senders (other receivers located or they fall outside of EAR), they perform random walk again. The number of failures in locating sources of signals after entering EAR of senders was also recorded. The bars represent means ± s.d. of 100,000 encounters.

The disadvantage of ballistic motion by signal senders in the presence of attracting signals arose in different forms depending on the locatability of receivers. High locatability (i.e., *p* = 1.0) resulted in long distances required for navigation to encounter senders (Fig. 3; Movie S1). On the other hand, low locatability (i.e., *p* = 0.5) required to try to locate senders more frequently, resulting in long distances of random walk to encounter senders (Fig. 3; Movie S2).

**Figure 4:**
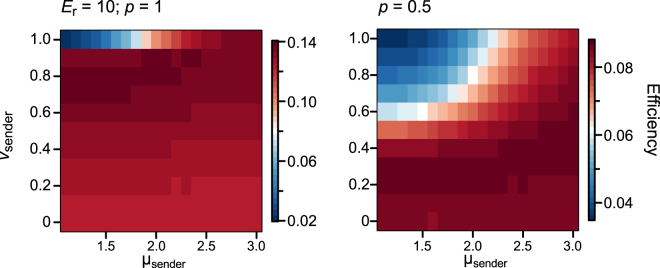
The optimal movements of signal senders with a given movement of receivers (μ_receiver_ = 1.1; *v*_receiver_ = 1; *L*^2^ = 100,000). The efficiency is computed as the average number of pairs encountered at a step.

Next, we explored the efficient movements of senders in the presence of attracting signals with the given movement patterns of receivers (μ_receiver_ = 1.1; *v*_receiver_ = 1). Under the same condition as the analysis described above, the optimal movements of senders were always slower and/or less diffusive than those of receivers (Fig. 4; Supplementary Figures S3, S4). In general, when the locatability of receivers was high (i.e., *p* = 1.0), slightly slow movements with relatively small µ values often achieved high efficiency (Fig. 4; Supplementary Figures S3, S4). When the locatability of receivers was low (i.e., *p* = 0.5), the movements which achieved high efficiency were slower and/or less diffusive compared to the case of high locatability (i.e., *p* = 1.0). This is because receivers faced difficulties to locate ballistic but slow senders, and tended to fall outside of EARs when the locatability of receivers was low (*p* = 0.5, Movie S3). On the other hand, when the locatability of receivers was high (*p* = 1.0), slower movements of senders compared to receivers were sufficient for receivers to locate senders (Movie S4).

**Figure 5:**
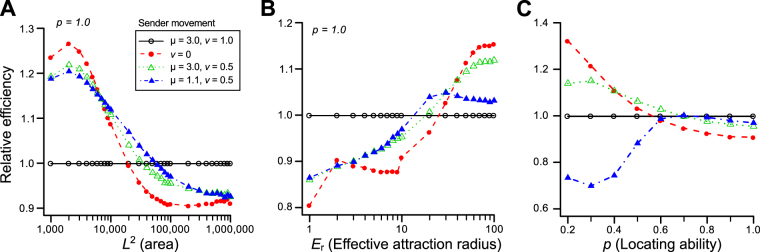
The effect of the searching area (*L*^2^_,_ A), the EAR of attracting signals (*E*_r,_ B) and the ability of receivers to locate senders (*p*_,_ C) on the efficiency of representative movements by signal senders and receivers with a fixed movement (μ_receiver_ = 1.1; *v*_receiver_ = 1). The efficiency is computed as the average number of pairs encountered at a step, where relative efficiency to sender with μ_sender_ = 3.0 and *v*_sender_ = 1 is described. Note that the absolute efficiencies increase with the increase of *E*_r_ or *p*, or decrease with the increase of *L*^2^. The results for senders with μ_sender_ = 1.1 and *v*_sender_ = 1 (same movement with receivers) are not shown, because their encounter rates are very low and visibility will become low. This combination always acquired the least searching efficiency except in the condition without attracting signals (i.e. *E*_r_ = 1.0).

The efficiency of movements of senders depended on the density of individuals (*n*/*L*^2^: the constant number of senders and receivers with the variety of the size of space), the size of EARs (*E*_r_), and the locatability of receivers (*p*). Under lower density (large *L*^2^), fast-moving senders covered a wider search area, which was the probable cause for the observed increase in efficiency (Fig. 5A). On the other hand, the slower-moving or even stationary senders achieved high efficiency under higher density (Fig. 5A). Similarly, as the EAR became larger, the optimal movements of senders became slower or even stationary ones (Fig. 5B). With the large EARs (e.g. *E*_r_ = 100), it was no longer difficult for receivers to enter the EARs. Under such conditions, the most important task for receivers is to locate senders more accurately. For the same reason, senders should stay and wait for receivers when the locatability of receivers was low (Fig. 5C).

## Discussion

We found that signal senders should move more slowly and/or less diffusively than receivers to improve mating encounters during mate search mediated by visual or acoustic attracting signals (Fig. 2). The mechanism underlying the results can be verbally understood as follows. In the presence of an attracting signal, the less diffusive senders are preferred by signal receivers, because receivers face difficulties to locate senders with ballistic motion. With high locatability of receivers (i.e., *p* = 1.0), receivers can exactly trace the movement of senders even if they are ballistic walkers (Movie S1). However, as ballistic senders often move straight in one direction, receivers cannot catch up with senders that move at the same speed as senders. This makes receivers take a long distance for navigation in order to encounter senders, which results in a low efficiency of encounters (Figs 2 and 3; Movie S1). On the other hand, with low locatability of receivers (i.e., *p* = 0.5), receivers cannot trace ballistic senders that rarely stay in a certain place. Even after receivers detect signals, they would fall easily outside the EAR of ballistic senders. This requires receivers to try to enter EAR repeatedly, which makes receivers take a long distance for a random walk to encounter senders and results in a low efficiency of encounters (Figs 2 and 3; Movie S2). Thus, senders need to move less diffusively than navigating receivers to utilize attracting signals more effectively.

Sexually dimorphic movements have been considered in the context of mate search^18,21^. A previous study reported that the highest encounter rate is achieved by sexual dimorphic movements when males search for moving females with Lévy flight, suggesting that observed interspecific variation in visually-cued mate search in butterflies can be understood in this framework^18^. Another study showed that sexually dimorphic movements evolve as a result of mutual optimization for mating encounters when individuals are in concentrated distribution under infinite and borderless searching space^21^. In the present study, we could add the advantages of sexually dimorphic movement patterns by focusing on the optimal movements for senders with visual or acoustic attracting signals to be found by signal receivers. We showed that sexually dimorphic movements can be adaptive in the presence of attracting signals even when individuals are present all over the searching space (i.e., periodic boundary conditions). These findings suggest that the sexual differentiation of movement patterns is likely to evolve in various species, calling for more empirical studies that separately analyze males’ and females’ movement data.

There are few studies that performed quantitative analyses on the sexual difference of movement patterns of animals, and our results are consistent with observations of representative species that use visual or acoustic attracting signals. For example, in field crickets, the best encounter efficiency is achieved when males call from a fixed position and females walk under low population density^23^. In most species of cicada, males make acoustical advertisements from perches and females approach them^35^. In some species, females respond by calling males with their wings flicking; males then make many short flight to form pairs^36^. Many nocturnal fireflies use bioluminescent signals, where flightless females glow slowly and attract flying males^27^.

Another possible example is swarming midges whose swarming is associated with mating. The location of their mates might be acoustically cued^37^. Previous studies have revealed that movement patterns of male midges during swarming are not ballistic but are indeed different types of Lévy walk, which might be accidental and be attributed to noise^38,39^. This finding appears inconsistent with our prediction that males (searchers or receivers) will perform ballistic motion (µ = 1.1). The discrepancy between model predictions and the observations may imply that optimality is not sufficient to understand the movement patterns of animals. More data of movement patterns from female midges are required to test our prediction that senders move more slowly or less diffusively than receivers.

The present model mainly considered the case of visual or acoustic signals, each of which has distinct characteristics of its availability. In the case of visual signals (e.g., light), the effective range should be shorter than that of acoustic signals, while locatability of signal receivers will be higher^33^. The difference between acoustic and visual signals can be interpreted as the difference of receiver’s locatability (*p*) or the effective range of signal (*E*_r_) in our simulations (Fig. 5). Thus, optimal movements of senders and receivers should differ between visual and acoustic signals even when the other conditions are identical. For example, under an identical density of individuals, we can expect that the degree of sexual dimorphism of movement patterns will be greater in species using acoustic signals than those using visual signals (Fig. 5). The acoustic communication might be found in nocturnal animals, whereas the visual communication in diurnal relatives. Note that sexually dimorphic movements will achieve the highest encounters irrespective of signal types if the signals attract receivers beyond simply enlarging the size of senders (Fig. 2).

As we simply approximated the attracting signals as EARs, there is a limitation to apply our model to most of the case of chemical signals. Navigation with olfactory sense is often performed in a prevailing wind (flow) and/or turbulence^29^. Therefore, animals should take these environmental factors into account to search for partners. For example, insects of the order Lepidoptera fly crosswind or downwind in the absence of an appropriate pheromone stimulus, which is an efficient search strategy to find odor plumes in wind^40,41^. Once they detect odor, receivers perform a variety of systematic searches upwind towards the source of odor^30,42^. Moreover, unlike acoustic and visual signals, chemical signals are not instantaneously effective because the molecules of odor need to be delivered to receivers^34^. If senders move fast, receivers face a problem to locate senders because the resulting time lag makes the signals inaccurate indicators of senders’ locations. Thus, much slower or stationary senders will be favored in the presence of time lags. In the case of chemical signals, our model assumption may be applied to the conditions under which odor only diffuses from the signal senders, which is expected in subterranean area or in proximity to the ground^31^. The case where receivers can be attracted to signal senders from only a short distance may also be possible. Mate search of termites might fulfill these conditions, which occurs on the ground. Female termites use sex pheromones to attract males and the effective radius is only a few centimeters in some species^43^.

The present study provides insights into strategies of effective attraction beyond the case of mate search. Visual attraction of phototactic insects is widely used in pest management^44^. Although their phototaxis is not for mate search, our results might be applied to light trapping techniques when the trap is regarded as signal sender. As far as we know, almost all of the light trapping devices for agricultural, orchard and forest pests are implemented as stationary traps. By exploring the optimal movements of the signal senders, our results imply the possibility of “moving traps” that need to move more slowly and/or less diffusively than the target insects.

Although we considered mate search that involves signal senders and receivers, similar asymmetry between searchers and targets can also occur in the case of foraging for food, where predators utilize visual cues to find and catch prey. Predators should optimize their movements so as to maximize encounters with prey, whereas prey should optimize their movements so as to minimize encounters with predators. For example, consider an initial condition where both predators and prey move with a Brownian walk at the same speed (µ_predator_ = µ_prey_ = 3), and the predators use a visual cue. This condition corresponds to our model with high locatability of receivers (*p* = 1.0). Here the Fig. 2C can be interpreted as fitness landscape for predators and inverse fitness landscape of prey. At the onset, movement strategies of predators are expected to evolve towards ballistic motion to catch their prey efficiently (µ_predator_ → 1.1), whereas no evolutionary change is expected for prey. Subsequently, prey will respond by evolving their movements towards ballistic motion to avoid predation by ballistic predators (µ_prey_ → 1.1). Finally, predators will counter-evolve their movements to enhance encounter rates with the ballistic prey. Thus, the coevolution of movement patterns of prey and predators is expected to result in µ_prey_ = 1.1 and µ_predator_ = 2.3, which should be an evolutionary stable state under this condition (Fig. 2C). Interestingly, this µ_predator_ value of Lévy walks mostly matches the case of marine predators that forage for moving prey^10^. As the slope towards this state is quite shallow, the realized coevolutionary state (or its fluctuation) would be affected by the population size, mutation rate and effects of mutations on the phenotype. Furthermore, it is possible to consider the reverse case, where prey can use visual cues to avoid predators (i.e., warning signals), as considered in a pursuit-and-evasion problems^45^. In the above consideration, a key point is that the coevolutionary processes between predators and prey shape the movement patterns of animals.

In conclusion, our study demonstrated that males and females should move differently to enhance encounter rates in the presence of attracting signals, where individuals of the signal-emitting sex should move less diffusively and/or more slowly. We also showed that the optimal extent of sexual dimorphism of movement patterns varies depending on searching circumstances, including the density of individuals, the effective range of signals, and the ability of receivers to locate senders. When we explore the random search strategies, targets such as prey, hosts, or mating partners have often been treated as if embedded in the environment^3^. However, these targets can also optimize their movement patterns for their own fitness benefits. The resulting mutual optimization of their movement patterns must have significant evolutionary consequence. Focusing not only on searchers but also on targets will lead to a better understanding of how animals move in nature and what is the optimal random search strategies for them.

## Models

We developed a simulation model where *n* signal senders and *n* receivers move to search for each other in a two dimensional periodic boundary condition (size: *L*^2^; Fig. 1A,B). Periodic boundary conditions are often used in the models or simulations of random search problems^13,14,17^, where an object passing through one side of the space will re-appear on the opposite side (i.e. moves on the surface of the torus). Individuals performed Lévy walks at a speed of *v*_receiver_ and *v*_sender_ until encountering an individual of the opposite sex. A Lévy walk was characterized by a uniform distribution for the turning angles [−π, π] and a power-law distribution for the move lengths. After generating a uniform random number *u* (0 < *u* ≤ 1), the step lengths were derived from the following equation: $l={l}_{0}{u}^{1/(1-{\rm{\mu }})}$, where *l*_0_ is the minimum move length and μ is the power-law exponent^16^. We set *l*_0_ as 1. To obtain a Lévy walk, once a move length and a direction were sampled from the respective distributions, the individuals walked in a straight line until reaching the specific move length or encountering a possible mating partner. In the simulations, it was assumed that all receivers and all senders had the same trait-value μ_receiver_ and μ_sender_.

For encounter events, receivers and senders were considered to have circular bodies (or distances short enough to encounter) with the same radius *r* (=0.5). When the distance between the centers of a receiver and a sender became smaller than 2*r* (=1.0), the two individuals encountered each other. As we assumed a monogamous mating scheme under which a receiver mates with only one sender (and vice versa), we considered that encountered pairs were removed from the searching arena. We did not consider the encounter between same-sex individuals. To make the density of individuals constant, we generated a new receiver and sender at a random position in the searching space after a pair disappeared. This treatment enabled us to obtain the average of the encounter rate, i.e., the number of pairs generated within a given time, as the measure of its efficiency.

In this study, we simply described that attracting signals were active within a space around senders with the effective attraction radius (EAR) to model the attraction of visual or acoustic signals. We assumed that senders had EARs denoted by *E*_r_ from the center of their body, within which receivers moved towards the senders with a probability of *p* and random direction with (1 − *p*) in each time step (Fig. 1C). Concretely, we considered that receivers were within EARs when the distance between the centers of a receiver and a sender became smaller than *E*_r_. Thus *E*_r_ = 1.0 corresponds to the conditions without EARs. When receivers failed to locate senders (the objective sender disappeared since another receiver had located it or the receiver fell outside of EAR), they performed random walk again. If a receiver detected more than one sender, it was assumed to orient toward the nearest sender.

In our simulations, the searching efficiency was measured as the average number of pairs encountered per time step with the fixed number of individuals *n* = 25. First, we examined the efficiency of the encounter when receivers’ and senders’ moving speeds were equal (*v*_receiver_ = *v*_sender_ = 1) by changing μ_receiver_ and μ_sender_ ranging from 1.1 to 3.0 by 0.1. To obtain the efficiencies, we performed simulation walks of 10,000,000 time steps. As signal receivers had two different phases of movements, we also calculated the distance traveled by signal receivers to encounter senders for random walk and navigation behavior respectively. We furthermore recorded the number of failures in locating senders after receivers entered EAR. In this analysis, the receivers of first encountered 100,000 pairs were examined because the number of pairs formed at 10,000,000 time step was different among combination of movement patterns of individuals. In addition, to confirm that the EAR can approximate the attracting signals rather than increase of the effective size of targets, we also conducted the simulations with the size of senders enlarged (*r*_sender_ = 9.5). The two individuals encountered each other when the distance between them became smaller than 10. Next, we fixed the movement patterns of receivers (*v*_receiver_ = 1; μ_receiver_ = 1.1), and examined the efficiency of random walk strategies for senders by changing *v*_senders_ from 0 to 1 by 0.05 and μ_senders_ from 1.1 to 3.0 by 0.1. We further analyzed the advantage of each movement pattern of senders across parameters (*L*^2^, *E*_r_ and *p*) by especially focusing on five movement patterns (*v*_senders_, μ_senders_) = (0, NA), (0.5, 1.1), (0.5, 3.0), (1.0, 1.1) and (1.0, 3.0) as the representative ones. Values and ranges of parameters are summarized in Table S1. The simulation program was implemented in Microsoft Visual Studio C++ 2012.

### Data availability

Source codes for all simulations are available in the Open Science Framework: osf.io/h45fb.

## Electronic supplementary material

Supplementary information

Movie S1

Movie S2

Movie S3

Movie S4

## References

[1] Bell WJ (1990). Searching behavior patterns in insects. Annu Rev Entomol.

[2] Viswanathan, G. M., Da Luz, M. G. E, Raposo, E. P. & Stanley, H. E. *The Physics of Foraging: An Introduction to Random Searches and Biological Encounters*. (Cambridge University Press., 2011).

[3] Bartumeus F, Catalan J (2009). Optimal search behavior and classic foraging theory. J Phys A Math Theor.

[4] Pyke GH, Pulliam HR, Charnov EL (1977). Optimal foraging: a selective review of theory and tests. Q Rev Biol.

[5] Humphries NE, Weimerskirch H, Queiroz N, Southall EJ, Sims DW (2012). Foraging success of biological Levy flights recorded *in situ*. Proc Natl Acad Sci USA.

[6] Raichlen DA, Wood BM, Gordon AD, Mabulla AZP, Marlowe FW (2013). Evidence of Lévy walk foraging patterns in human hunter-gatherers. Proc Natl Acad Sci USA.

[7] Reynolds, A. M. & Frye, M. A. Free-flight odor tracking in *Drosophila* is consistent with an optimal intermittent scale-free search. *PLoS One* **2** (2007). 10.1371/journal.pone.0000354 PMC1831497 17406678

[8] Reynolds AM, Smith A, Reynolds DR, Carreck NL, Osborne JL (2007). Honeybees perform optimal scale-free searching flights when attempting to locate a food source. J Exp Biol.

[9] Reynolds AM (2007). Displaced honey bees perform optimal scale-free search flights. Ecology.

[10] Sims DW (2008). Scaling laws of marine predator search behaviour. Nature.

[11] Sims DW (2014). Hierarchical random walks in trace fossils and the origin of optimal search behavior. Proc Natl Acad Sci USA.

[12] Viswanathan GM (1996). Lévy flight search patterns of wandering albatrosses. Nature.

[13] Viswanathan GM (1999). Optimizing the success of random searches. Nature.

[14] Bartumeus F, Da Luz MGE, Viswanathan GM, Catalan J (2005). Animal search strategies: a quantitative random walk analysis. Ecology.

[15] Reynolds AM, Bartumeus F (2009). Optimising the success of random destructive searches: Lévy walks can outperform ballistic motions. J Theor Biol.

[16] Bartumeus F, Catalan J, Fulco U, Lyra M, Viswanathan GM (2002). Optimizing the encounter rate in biological interactions: Lévy versus Brownian strategies. Phys Rev Lett.

[17] James A, Plank MJ, Brown R (2008). Optimizing the encounter rate in biological interactions: Ballistic versus Lévy versus Brownian strategies. Phys Rev E.

[18] Reynolds AM (2006). Optimal scale-free searching strategies for the location of moving targets: New insights on visually cued mate location behaviour in insects. Phys Lett A.

[19] Abe MS, Shimada M (2015). Lévy walks suboptimal under predation risk. PLoS Comput Biol.

[20] Palyulin VV, Chechkin AV, Metzler R (2014). Levy flights do not always optimize random blind search for sparse targets. Proc Natl Acad Sci USA.

[21] Mizumoto N, Abe MS, Dobata S (2017). Optimizing mating encounters by sexually dimorphic movements. J R Soc Interface.

[22] Yoshida K, Iwasa Y (2013). The evolution of sex differences in mate-attracting signalling. Evol Ecol Res.

[23] Hissmann K (1990). Strategies of mate finding in the European field cricket (*Gryllus campestris*) at different population densities: a field study. Ecol Entomol.

[24] Hunt RE, Nault LR (1991). Roles of interplant movement, acoustic communication, and phototaxis in mate-location behavior of the leafhopper *Graminella nigrifrons*. Behav Ecol Sociobiol.

[25] Snedden WA, Sakaluk SK (1992). Acoustic signalling and its relation to male mating success in sagebrush crickets. Anim Behav.

[26] Holwell GI, Barry KL, Herberstein ME (2007). Mate location, antennal morphology, and ecology in two praying mantids (Insecta: Mantodea). Biol J Linn Soc.

[27] Lewis SM, Cratsley CK (2008). Flash signal evolution, mate choice, and predation in fireflies. Annu Rev Entomol.

[28] El-Sayed, A. M. The Pherobase: database of pheromones and semiochemicals. at http://www.pherobase.com/ (2016).

[29] Wyatt, T. D. *Pheromones and Animal Behavior: Chemical Signal and Signatures*. (Cambridge University Press, 2014).

[30] Cardé RT, Willis MA (2008). Navigational strategies used by insects to find distant, wind-borne sources of odor. J Chem Ecol.

[31] Reynolds AM (2008). Deterministic walks with inverse-square power-law scaling are an emergent property of predators that use chemotaxis to locate randomly distributed prey. Phys Rev E.

[32] Bau, J. & Cardé, R. T. Modeling optimal strategies for finding a resource-linked, windborne odor plume: Theories, robotics, and biomimetic lessons from flying insects. In *Integrative and Comparative Biology* **55**, 461–477 (Oxford University Press, 2015). 10.1093/icb/icv036 25980569

[33] Alcock, J. *Animal Behaviour*. *An Evolutionary Approach*. (Mass.: Sinauer Associates, 1989).

[34] Wyatt, T. D. *Pheromones and Animal Behaviour: Communication by Smell and Taste*. (Cambridge University Press, 2003).

[35] Cooley JR (2001). Long-range acoustical signals, phonotaxis, and risk in the sexual pair-forming behaviors of *Okanagana canadensis* and *O rimosa* (Hemiptera: Cicadidae). Ann Entomol Soc Am.

[36] Gwynne DT (1987). Sex-biased predation and the risky mate-locating behaviour of male tick-tock cicadas (Homoptera: Cicadidae). Anim Behav.

[37] Gorbonos D (2016). Long-range acoustic interactions in insect swarms: An adaptive gravity model. New J Phys.

[38] Reynolds AM, Ouellette NT (2016). Swarm dynamics may give rise to Lévy flights. Sci Rep.

[39] Reynolds AM, Sinhuber M, Ouellette NT (2017). Are midge swarms bound together by an effective velocity-dependent gravity?. Eur Phys J E.

[40] Sabelis MW, Schippers P (1984). Variable wind directions and anemotactic strategies of searching for an odour plume. Oecologia.

[41] Dusenbery DB (1990). Upwind searching for an odor plume is sometimes optimal. J Chem Ecol.

[42] Mafra-Neto A, Cardé RT (1994). Fine-scale structure of pheromone plumes modulates upwind orientation of flying moths. Nature.

[43] Bordereau, C. & Pasteels, J. M. In *Biology of Termites: A Modern Synthesis* 279–320 10.1007/978-90-481-3977-4_11 (Springer Netherlands, 2011).

[44] Shimoda M, Honda K (2013). Insect reactions to light and its applications to pest management. Applied Entomology and Zoology.

[45] Oshanin G, Vasilyev O, Krapivsky PL, Klafter J (2009). Survival of an evasive prey. Proc Natl Acad Sci USA.

