# Supplementary material for: The optimal movement patterns for mating encounters with sexually asymmetric detection ranges
Source: Sci Rep. 2018 Feb 20;8:3356. doi: 10.1038/s41598-018-21437-3 (PMC5820271; doi:10.1038/s41598-018-21437-3)
Supplement: Supplementary file 1 — Supplementary information [file 41598_2018_21437_MOESM1_ESM.pdf]

Supplementary information for

The optimal movement patterns for mating encounters with sexually asymmetric detection ranges

Nobuaki Mizumoto*[*] and Shigeto Dobata

Laboratory of Insect Ecology, Graduate School of Agriculture, Kyoto University, Kitashirakawa-oiwakecho, Sakyo-ku, Kyoto 606-8502, Japan.

N. M. (*correspondence): mizumoto.nobuaki.75a@st.kyoto-u.ac.jp
nobuaki.mzmt@gmail.com
S. D.: dobata@kais.kyoto-u.ac.jp

NM: 0000-0002-6731-8684
SD: 0000-0003-1586-6758

Contents:

Table S1
Fig. S1-S4
Movie S1-S4

Table S1. Notations used in this manuscript

		Value(s) or range in	
**Symbol**	**Definition**	**Same speed**	**Fixed receivers**
$\boldsymbol{n}$	Number of senders or receivers	25	25
$\boldsymbol{L^2}$	Size of searching space	10^4, 10^5, 10^6	10^5 or [10^4–10^6]
$\boldsymbol{v_{receiver}}$	Speed of receivers	1	1
$\boldsymbol{v_{sender}}$	Speed of senders	1	[0–1]
$\boldsymbol{l_0}$	Minimum move length	1	1
$\boldsymbol{\mu_{receiver}}$	Power-law exponent of receivers	[1.1–3.0]	1.1
$\boldsymbol{\mu_{sender}}$	Power-law exponent of senders	[1.1–3.0]	[1.1–3.0]
r	Size of individuals	0.5, 9.5	0.5
$\boldsymbol{E_r}$	EAR of attracting signal	1, 10	10 or [1–200]
$\boldsymbol{p}$	Probability to orient within EAR	0.5, 1	0.5, 1 or [0.2–1.0]

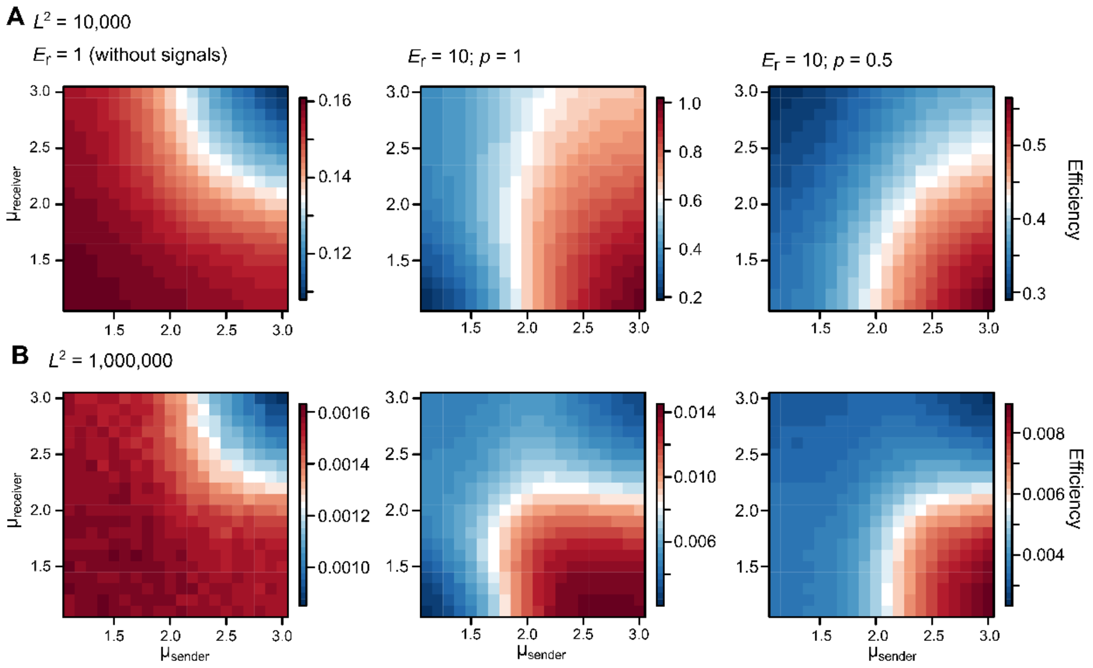

Figure S1: The simulation results performed with receivers and senders with the same moving velocity ($v_{receiver}$ = v_{sender} = 1) across the different density of individuals (A: L^2 = 10,000; B: L^2 = 1,000,000).The results were qualitatively similar with Figure 2 (L^2 = 100,000).For these results, we performed simulation walks of 10,000,000.

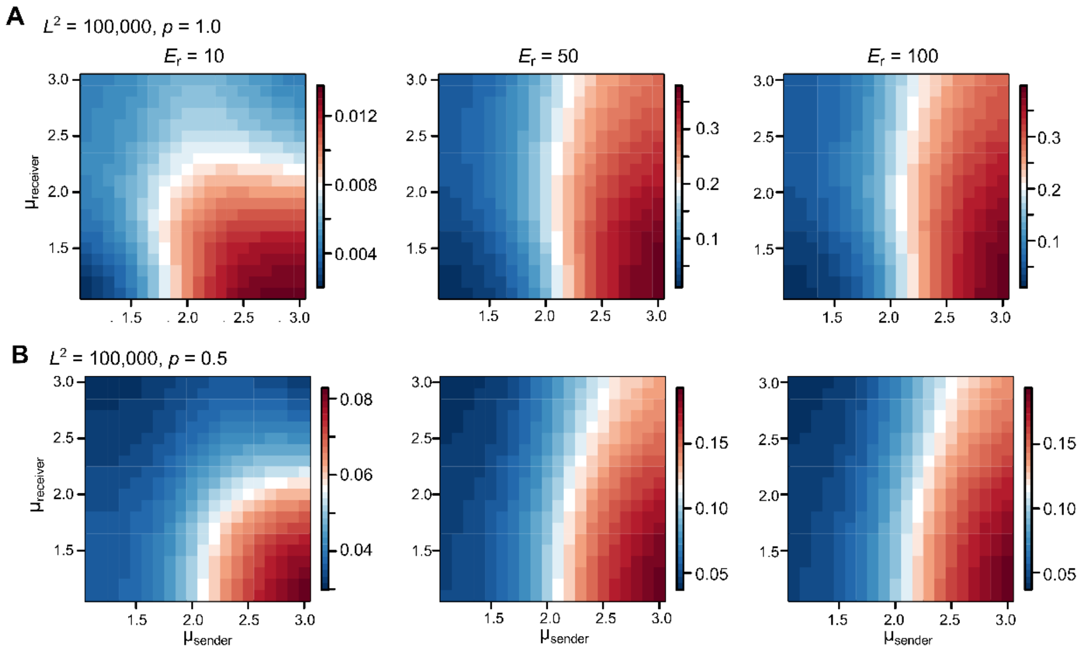

Figure S2: The simulation results performed with receivers and senders with the same moving velocity ($v_{receiver}$ = v_{sender} = 1) across the different size of EAR (L^2 = 100,000).For these results, we performed simulation walks of 10,000,000.

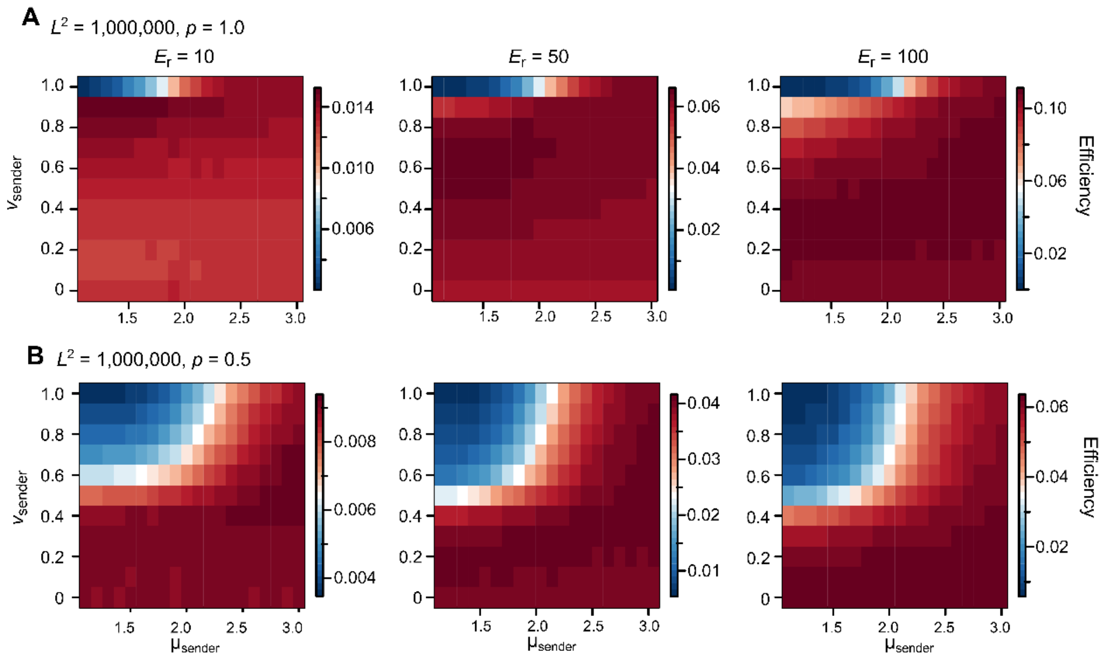

Figure S3: The effect of the effective attraction radius (E_r) of attracting signals on the optimal movements of senders with a given movements of receivers ($\mu_{receiver}$ = 1.1; $v_{receiver}$ = 1). The efficiency is computed as the average number of pairs encountered at a step. For these results, we performed simulation walks of 10,000,000.

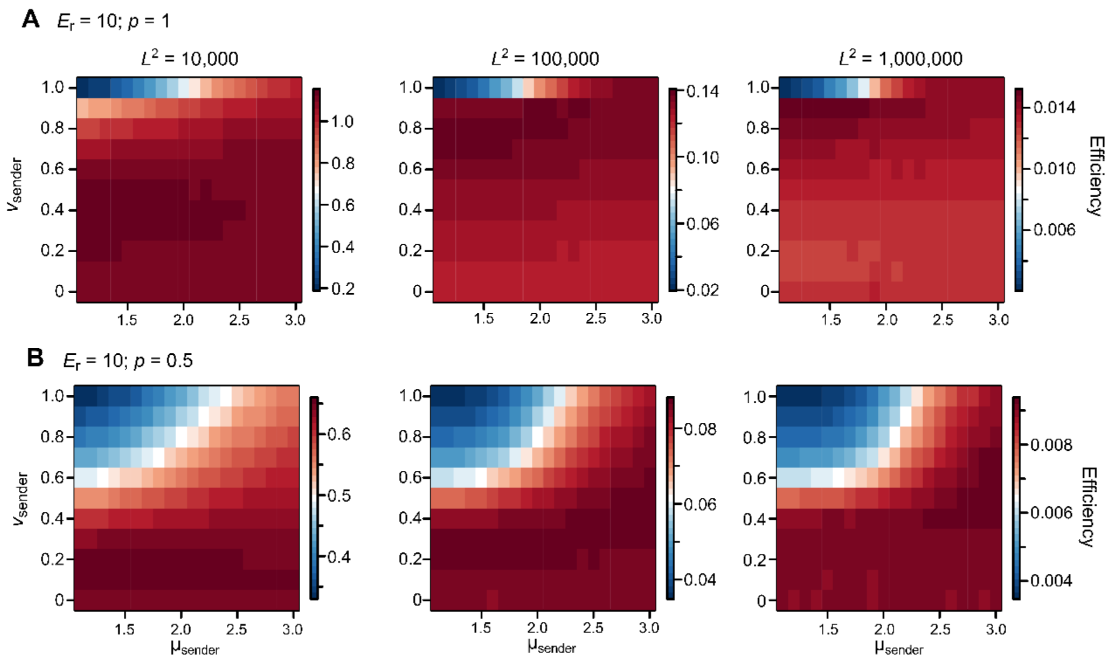

Figure S4: The effect of the size of searching space (L^2) on the optimal movements of senders with a given movement of receivers ($\mu_{receiver}$ = 1.1; $v_{receiver}$ = 1.0). The efficiency is computed as the average number of pairs encountered at a step. For these results, we performed simulation walks of 10,000,000.
